# Inhibition of *α*-Glucosidase by Thiosulfinate as a Target for Glucose Modulation in Diabetic Rats

**DOI:** 10.1155/2016/7687915

**Published:** 2016-03-09

**Authors:** Abdulrahman L. Al-Malki

**Affiliations:** Department of Biochemistry, Faculty of Science, Bioactive Natural Products Research Group and Experimental Biochemistry Unit, King Fahd Medical Research Center, King Abdulaziz University, P.O. Box 80203, Jeddah 21589, Saudi Arabia

## Abstract

Postprandial hyperglycemia is a predisposing factor for vascular dysfunction and organ damage. *α*-glucosidase is a hydrolytic enzyme that increases the glucose absorption rate and subsequently elevates blood glucose levels. Garlic (*Allium sativum* L.) is a rich source of several phytonutrients, including thiosulfinate (THIO). The aim of this study was to evaluate the ability of THIO, a potent inhibitor of intestinal *α*-glucosidase, to reduce postprandial blood glucose. Male albino rats were randomly assigned to five different groups (*n* = 10/group). Group 1 served as the control group. Groups 2–5 were injected intraperitoneally with a single dose of streptozotocin (STZ) to induce diabetes. Group 2 comprised untreated diabetic rats. Groups 3 and 4 contained diabetic rats that were given THIO orally (20 mg/kg body weight/day and 40 mg/kg body weight/day, resp.). Group 5 was the positive control having diabetic rats treated orally with acarbose (10 mg/kg body weight/day; positive control). Diabetic rats treated with THIO displayed a significant blood glucose reduction (*p* < 0.001 and < 0.01 by analysis of variance, resp.) and a significant elevation in insulin compared with that of untreated rats. THIO is an effective noncompetitive intestinal *α*-glucosidase inhibitor that promotes hypoglycemic action (*p* < 0.001) in STZ-injected rats. THIO is a promising agent for the management of postprandial hyperglycemia.

## 1. Introduction

Sustained hyperglycemia is a predisposing factor for microvascular dysfunction, neural complications, and organ damage [1–3]. Morbidity and mortality rates in patients with diabetes have increased [4]. Chronic blood glucose fluctuations increase peripheral neuron oxidative damage because of the elevation of nonenzymatic reactions with functional proteins in vital organs, such as the kidneys and connective tissue in the retina [5]. Prolonged hyperglycemia induces polyol production in vital organs [6]. *α*-glucosidase is a hydrolytic enzyme released from intestinal mucosal cells that breaks down polysaccharides to monosaccharides and increases glucose absorption, subsequently raising blood glucose levels. *α*-glucosidase suppression is a target for delaying sugar digestion and reducing glucose absorption and subsequently postprandial insulin release [7].

Acarbose is a complex oligosaccharide that delays the digestion and absorption of dietary carbohydrates, thereby decreasing postprandial hyperglycemia. In contrast to sulfonylureas, acarbose does not enhance insulin secretion. In fact, acarbose is an established *α*-glucosidase enzyme inhibitor which is found to be effective in antidiabetic treatment and to reduce the risk of cardiovascular disease [8, 9]. The antihyperglycemic action of acarbose results from competitive, reversible inhibition of pancreatic *α*-amylase and membrane-bound intestinal *α*-glucosidase [10]. *α*-glucosidase inhibitors can be used for the management or treatment of these complications [11]. The current clinical trend is the utilization of naturally active compounds as replacement therapies [12, 13].

Thiosulfinate (THIO) is an active ingredient in garlic (*Allium sativum*) and onion (*A. cepa*) which is used as a food additive and in active food packaging [14, 15]. Onion is rich in THIO and organosulfur compounds [16]. Thiosulfinates were reported to be lacking radical-trapping potential in liposomes and cells; nevertheless, thiosulfinates and their derivative showed diverse antioxidant potential in different experiments [17, 18]. THIO produces eye-irritation and stimulates the lacrimal glands. Furthermore, it has been used as a complementary therapy to treat many diseases and conditions, including diarrhea, headache, and flu-like symptoms [19]. These biological activities are related to THIO, volatile sulfur compounds that are responsible for the pungency of these vegetables. It is worth mentioning that THIO or alkane(ene) thial-*S*-oxide formation is catalyzed by the enzyme alliinase from their respective* S*-alk(en)yl cysteine sulfoxides [20]. These compounds are not present in the intact bulbs but are formed by enzymatic reaction of the precursor [21]. Keeping in view the use of herbal drug products worldwide, there is a need to explore activity and toxicity of natural drugs. Both* A. sativum* and* A. cepa* are well-investigated plants possessing healing potential with low toxicity; however, the pure compounds isolated from these plants and their derivatives need further investigation [22, 23]. There have been no previous reports investigating possible intestinal *α*-glucosidase activity inhibition by THIO or evaluating the hypoglycemic potential of THIO. This study was designed to evaluate THIO as a potential inhibitory agent of *α*-glucosidase. Ultimately, THIO treatment could be used to manage blood glucose levels.

## 2. Experimental Section

### 2.1. Chemicals

All chemicals, including THIO, streptozotocin (STZ), p-nitrophenyl glucopyranoside (PNPG), acarbose, and phosphate-buffered saline, are ultrapure grade and were obtained from Bio-Rad (Mumbai, India).

### 2.2. Animals

Fifty male albino rats weighing 100–120 g were used in this study. Animals were housed under standard conditions and received a standard pellet diet and water* ad libitum*. All animal protocols were approved by King Abdulaziz University Animal Ethics Committee. The animals were divided into five groups of 10 animals each. Group 1 was given sucrose (2 g/kg body weight) orally and it served as a control group. Rats in Groups 2–5 were injected intraperitoneally (i.p.) with a single dose of STZ (65 mg/kg body weight) to induce diabetes [24]. Animals with fasting blood glucose levels above 250 mg/dL were considered diabetic. Group 2 consisted of untreated diabetic rats. Groups 3 and 4 comprised diabetic rats that were given sucrose (2 g/kg) orally and THIO (20 and 40 mg/kg body weight/day, resp.) in phosphate-buffer saline (pH 7.2). Group 5 consisted of diabetic rats given sucrose and acarbose (10 mg/kg body weight/day). The selected THIO dosages were determined to be safe based on previous studies and equivalency to human dosages [18]. In addition, blood glucose levels were determined by glucometer at 0, 30, 60, and 120 minutes after glucose loading. Four weeks after treatment, animals were fasted overnight (12 h) and lightly anesthetized with 10% thiopental. Blood was collected directly from the animals and processed to get serum. Serum was stored at −80°C for analysis of insulin.

### 2.3. Oral Glucose Tolerance (OGT) Testing

Another ten diabetic rats were grouped into two groups of five animals each. Animals were fasted for 12 h. Group 1 (the control) was given glucose orally (2 g/kg body weight). Group 2 was given glucose (2 g/kg body weight) and THIO (20 mg/kg body weight). Blood glucose was measured at 0, 30, 60, and 120 minutes after glucose administration. The change in blood glucose following oral loading was used for analysis.

### 2.4. Determination of Serum Insulin

Serum insulin was determined by using the Insulin ELISA kit (enzyme linked immunosorbent assay) obtained from Bio-Rad, England.

### 2.5. Determination of Intestinal *α*-Glucosidase Activity

A portion of the small intestine (200 mg) was extracted in 4 mL of 50 mM cold phosphate buffer (pH 7.3) and sonicated for 15 min at 4°C. After vigorous vortexing for 20 min, the suspension was centrifuged at 8,000 ×g at 4°C for 20 min. The resulting supernatant was used for the intestinal *α*-glucosidase activity assay. A reaction mixture containing 50 *μ*L of phosphate buffer (50 mM; pH 6.8) and 20 *μ*L of 1 mM PNPG was added as the substrate. Following incubation at 37°C for 30 min, the reaction was terminated by adding 50 *μ*L of sodium carbonate (0.25 M). Acarbose was used as a positive control and water as a negative control. Enzymatic activity was quantified by measuring the absorbance at 410 nm. Inhibition (%) was calculated as [(Ac − As)/Ac] × 100, where Ac and As are the absorbance of the control and the experimental condition, respectively. *α*-glucosidase inhibition kinetics were measured using serial concentrations of PNPG (0.2–1 mM) as the substrate [19]. The inhibition type was determined by Lineweaver-Burk plot analysis of the data, calculated using Michaelis-Menten kinetics.

### 2.6. Statistical Analysis

Experiments were performed in duplicate and results were expressed as mean ± SD. Data were analyzed by analysis of variance. A value of *p* < 0.05 was considered significant.

## 3. Results

**Figure 1:**
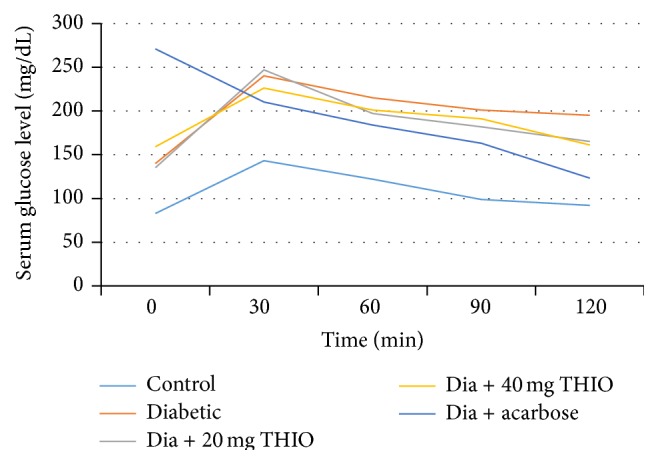
Glucose tolerance curve following oral glucose tolerance testing in rats. Control rats (Group 1, light blue), diabetic rats (Group 2, orange), diabetic rats treated with THIO (Group 3, 20 mg/kg body weight, gray; Group 4, 40 mg/kg body weight, yellow), or diabetic rats treated with acarbose (Group 5, blue). Results are expressed as mean ± SD.

The glucose tolerance curve in diabetic rats treated with THIO (20 or 40 mg/kg body weight) was compared with the control and displayed in Figure 1. Plasma glucose levels in the control rats reached a peak 30 min after oral glucose administration and gradually decreased to baseline within 90 min (Figure 1). Blood glucose levels in diabetic rats treated with THIO (20 or 40 mg/kg body weight) were significantly lower than those of the diabetic untreated group at 30, 60, and 120 min following glucose administration. The area under the curve during the OGT test was significantly decreased following THIO treatment (Figure 1).

Acarbose treatment (10 mg/kg body weight) resulted in a significant reduction in plasma glucose levels at 30, 60, 120, and 180 min after oral glucose administration.

**Figure 2:**
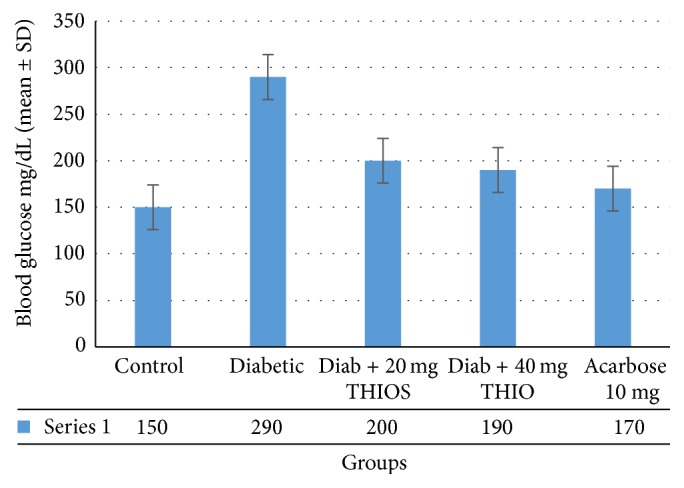
Blood glucose level change from baseline following sucrose loading and THIO administration. The effect of THIO (20 and 40 mg/kg body weight orally) or acarbose (10 mg/kg body weight; positive control) on blood glucose levels at 30 minutes after glucose administration compared with that of diabetic and control rats is displayed.

**Figure 3:**
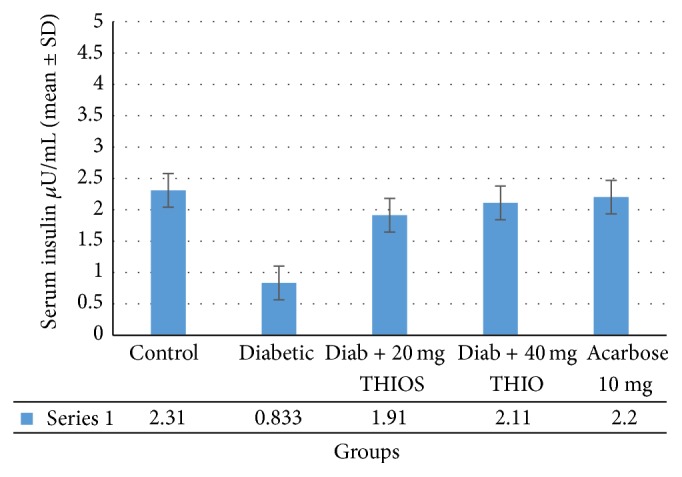
Fasting serum insulin level in different groups at the end of 4 weeks (mean ± SD).

Postprandial blood glucose levels were measured following glucose administration in rats (Figure 2). In the control group, blood glucose levels increased by an average of 150 mg/dL after 30 minutes. In diabetic rats after 30 minutes, glucose levels were 290 mg/dL. Diabetic rats treated with THIO (20 or 40 mg/kg body weight) or acarbose (10 mg/kg body weight) displayed a significant and dose dependent reduction of blood glucose compared with untreated rats (*p* < 0.001). Blood glucose levels did not differ significantly between diabetic rats treated with acarbose and those treated with THIO (20 or 40 mg/kg body weight). Results obtained (Figure 3) showed that serum insulin of diabetic rats was significantly (*p* < 0.001) decreased (0.83 ± 0.23 *μ*U/mL) as compared with the control group (2.31 ± 0.42 *μ*U/mL). Diabetic rats were treated with THIO (20 or 40 mg/kg body weight). Acarbose treatment showed insulin level to be 1.91 ± 0.42 *μ*U/mL, 2.11 ± 0.36 *μ*U/mL, and 2.20 ± 0.29 *μ*U/mL, respectively.

**Figure 4:**
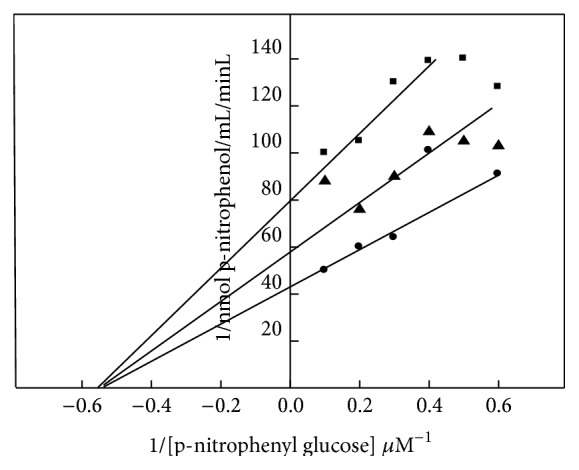
Mechanism of *α*-glucosidase inhibition following THIO treatment. Lineweaver-Burk plot of *α*-glucosidase inhibition by THIO. *α*-glucosidase was incubated with varying p-nitrophenyl glucopyranoside (PNPG) concentrations (−0.6–0.6 *μ*M) in the presence of THIO (0.25, 0.5, or 1 mg/mL) following incubation at 37°C for 30 min.

The mechanism by which THIO inhibited intestinal *α*-glucosidase activity was analyzed using Lineweaver-Burk double reciprocal curves. The results revealed a noncompetitive inhibition of enzyme activity (Figure 4). *K*_*m*_ values were unchanged while *V*_max_ was significantly decreased in rats treated with THIO at different concentrations compared with that of untreated diabetic rats. Acarbose treatment resulted in greater *α*-glucosidase inhibition than that following THIO treatment.

## 4. Discussion

The literature review revealed that patients with untreated diabetes are at risk for developing macrovascular and microvascular complications, including retinopathy, nephropathy, neuropathy, and cardiovascular diseases [8, 9]. Postprandial hyperglycemia increases the risk of diabetic complications. High intake of carbohydrates, which are rapidly hydrolyzed into absorbable monosaccharides by *α*-glucosidase, can cause a rise in blood glucose levels [21]. In the present study *α*-glucosidase enzyme was challenged using rats as experimental modal leading to promising results which were full in agreement with the earlier work [1–3]. However, attempts were made to investigate the mechanism of action of the activity observed.

Antidiabetic action of garlic is well identified. The hypoglycemic effects of garlic are attributed to the presence of active sulfur compounds like THIO. Garlic and garlic extracts have been shown to be effective in reducing insulin resistance and affecting the levels of other hormones [19] keeping in view the common use of garlic and the importance of THIO in controlling diabetic complications and the mechanism of action is not clear. The current study was undertaken to know the possible mechanism of THIO as postprandial management. The postprandial glucose reduction observed following THIO treatment is primarily due to the inhibition of intestinal glucose absorption well explored. THIO treatment significantly reduced the postprandial hyperglycemia associated with glucose and sucrose loading, as a result of inhibition of *α*-glucosidase activity and intestinal glucose absorption.

The results of the present study add further support to the earlier evidence based reports. It was observed that the control group animals in the present experiment displayed increased blood glucose levels 2 hours after sucrose loading. The suppression of postprandial glucose levels in diabetic rats could be due to THIO inhibition of sucrose digestion in the small intestine. This is consistent with a previous study that stated that THIO, arising from* Allium* species, possessed hypoglycemic action in diabetic animals [25]. The observed postprandial glucose reduction may be due to decreased glucose absorption following THIO treatment. To support the results obtained the effects of THIO on glucose loading in normal and diabetic rats were investigated.

THIO given orally (20 mg/kg body weight/day and 40 mg/kg body weight/day) exhibited a significant management of blood glucose and insulin. The results of the present study very well indicated that oral treatment with THIO at different dose levels (20 and 40 mg/kg body weight daily) induced a statistically significant hypoglycemic effect by stimulating the *β*-cells of pancreas. THIO was found to control the blood glucose and insulin in STZ induced diabetic rats leading to glucose homeostasis. Hence we concluded that THIO treatment possessed postprandial regulation of blood glucose property by stimulating the insulin secretion from the pancreatic *β*-cells and its management of diabetic complications.

The ability of THIO to inhibit *α*-glucosidase was evaluated using yeast *α*-glucosidase and mammalian *α*-glucosidase, which are commonly used for investigating Lineweaver-Burk *α*-glucosidase inhibitors from microbes and medicinal plants [26]. In the present study results it was observed that THIO displayed noncompetitive inhibition of *α*-glucosidase, as indicated by a decreased *V*_max_ and an unchanged *K*_*m*_.

*α*-glucosidase activity was dose-dependently reduced by THIO (Figure 3). THIO may bind to *α*-glucosidase active sites, and the inhibitory action of THIO was increased by increasing concentrations. Double reciprocal plots of *α*-glucosidase with THIO revealed that the mechanism of enzyme inhibition was noncompetitive. The relationship between oxidative stress and diabetes development, in which free radicals and reactive oxygen species are involved, is complex [27]. Nevertheless, the results of the current experiment showed THIO treatment to be primarily responsible for antioxidant effects [28, 29]. These results added support to the current findings to conclude that the antidiabetic effect of THIO might be due to its free radical scavenging potential, which prevented STZ induced oxidative damage on the pancreatic *β*-cell and the subsequent loss of insulin synthesis and secretion.

## 5. Conclusions

THIO displayed a noncompetitive inhibitory effect on the enzyme *α*-glucosidase, resulting in effective postprandial glucose suppression. In addition, it stimulates insulin release from pancreas, for THIO could be used to treat postprandial hyperglycemia and protect against diabetic complications.
